# Effect of Psychological Intervention on Fear of Cancer Recurrence: A Systematic Review and Meta-Analysis

**DOI:** 10.1200/JCO.19.00572

**Published:** 2019-09-18

**Authors:** Nina M. Tauber, Mia S. O’Toole, Andreas Dinkel, Jacqueline Galica, Gerry Humphris, Sophie Lebel, Christine Maheu, Gozde Ozakinci, Judith Prins, Louise Sharpe, Allan “Ben” Smith, Belinda Thewes, Sébastien Simard, Robert Zachariae

**Affiliations:** ^1^ Aarhus University, Aarhus, Denmark; ^2^ International Psycho-Oncology Society Fear of Cancer Recurrence Special Interest Group, Toronto, Ontario, Canada; ^3^ Technical University of Munich, Munich, Germany; ^4^ Queen’s University, Kingston, Ontario, Canada; ^5^ University of St Andrews, St Andrews, United Kingdom; ^6^ University of Ottawa, Ottawa, Ontario, Canada; ^7^ McGill University, Montréal, Québec, Canada; ^8^ Radboud University Medical Centre, Nijmegen, the Netherlands; ^9^ University of Sydney, Sydney, NSW, Australia; ^10^ Ingham Institute for Applied Medical Research and University of New South Wales, Sydney, NSW, Australia; ^11^ Université du Québec à Chicoutimi, Saguenay, Québec, Canada; ^12^ Aarhus University Hospital, Aarhus, Denmark

## Abstract

**PURPOSE:**

Fear of cancer recurrence (FCR) is a significantly distressing problem that affects a substantial number of patients with and survivors of cancer; however, the overall efficacy of available psychological interventions on FCR remains unknown. We therefore evaluated this in the present systematic review and meta-analysis.

**METHODS:**

We searched key electronic databases to identify trials that evaluated the effect of psychological interventions on FCR among patients with and survivors of cancer. Controlled trials were subjected to meta-analysis, and the moderating influence of study characteristics on the effect were examined. Overall quality of evidence was evaluated using the GRADE system. Open trials were narratively reviewed to explore ongoing developments in the field (PROSPERO registration no.: CRD42017076514).

**RESULTS:**

A total of 23 controlled trials (21 randomized controlled trials) and nine open trials were included. Small effects (Hedges’s *g*) were found both at postintervention (*g* = 0.33; 95% CI, 0.20 to 0.46; *P* < .001) and at follow-up (*g* = 0.28; 95% CI, 0.17 to 0.40; *P <* .001). Effects at postintervention of contemporary cognitive behavioral therapies (CBTs; *g* = 0.42) were larger than those of traditional CBTs (*g* = 0.24; β = .22; 95% CI, .04 to .41; *P* = .018). At follow-up, larger effects were associated with shorter time to follow-up (β = −.01; 95% CI, −.01 to −.00; *P* = .027) and group-based formats (β = .18; 95% CI, .01 to .36; *P* = .041). A GRADE evaluation indicated evidence of moderate strength for effects of psychological intervention for FCR.

**CONCLUSION:**

Psychological interventions for FCR revealed a small but robust effect at postintervention, which was largely maintained at follow-up. Larger postintervention effects were found for contemporary CBTs that were focused on processes of cognition—for example, worry, rumination, and attentional bias—rather than the content, and aimed to change the way in which the individual relates to his or her inner experiences. Future trials could investigate how to further optimize and tailor interventions to individual patients’ FCR presentation.

## INTRODUCTION

Despite improved treatments and prognoses, many survivors of cancer face the possibility that their cancer may return. For some, uncertainty leads to high levels of fear of cancer recurrence (FCR), which is defined as the “fear, worry, or concern about cancer returning or progressing.”^1(p424)^ Individuals with active disease may fear that stable disease will progress, and survivors of cancer have been found to fear recurrence after completion of active treatment.^2^ Such fears and worries can thus be present from the beginning of diagnosis and continue throughout treatment and the survivorship trajectory. It is common to experience some degree of FCR, and transitory or low levels of FCR may even be adaptive, alerting the patient to signs of new or recurring cancer and encouraging positive health behaviors.^3,4^ Persistent and excessive fear, however, can be highly debilitating.^1,2,5^

FCR is among the most commonly reported concerns by survivors of cancer and often their most frequently endorsed unmet need.^6^ A comprehensive review^2^ estimates that, across different cancers, 22% to 87% of survivors of cancer report moderate to high FCR, and 0% to 15% report high or clinical levels of FCR, although there currently is no agreed upon clinical cutoff. Furthermore, FCR seems to remain relatively stable over time.^2,7^ Associations have been reported between FCR and depression, poorer quality of life, and impaired functioning,^4,8^ and a growing body of evidence suggests that people with high FCR may both overuse health services and avoid appropriate tests to identify recurrence in a timely fashion.^9^ These results emphasize the need for effective, evidence-based treatments for FCR.

Interventions for FCR are emerging and the number of randomized controlled trials (RCTs) that have evaluated such interventions is expanding rapidly. A recent review^10^ identified five RCTs of FCR interventions that were published in 2016 and 2017 alone, and several study protocols and feasibility studies have been published during this period.^11-18^ The exact number of existing psychological interventions for FCR has not been systematically identified, and little is known about their efficacy in alleviating FCR symptoms. Thus far, only one meta-analytical evaluation of the effect of mind–body interventions on FCR and cancer-related uncertainty in 19 RCTs has been published,^19^ which reported a small effect both at postintervention (Hedges’s *g* = −0.36; *P* < .001) and at follow-up (*g* = −0.31; *P* < .001). However, this study included not only psychological interventions, but also physical interventions—for example, yoga or dance. Second, only 13 of the 19 studies included an FCR-specific measure, with the remaining studies assessing more general cancer-related uncertainty. Although cancer-related uncertainty overlaps with FCR,^20-23^ uncertainty does not necessarily pertain to the perceived risk of recurrence or progression, but can also relate to other issues that are associated with cancer diagnosis and treatment, including work-related issues or symptom management. Third, potentially important between-study differences remained unexplored in the former review,^19^ including the type of psychotherapeutic framework and whether the intervention specifically targeted FCR. Finally, the number of FCR interventions being developed and evaluated is rapidly expanding, and not all relevant studies were included in the former review. Taken together, attempts to synthesize the literature on psychological interventions for FCR are limited, and an up-to-date review of current developments in the field is lacking.^1^

The primary objective of the current study was to conduct a systematic review and meta-analysis of the efficacy of psychological interventions for alleviating FCR among patients with and survivors of cancer as evaluated in controlled trials. We hypothesized that psychological interventions are efficacious in reducing FCR symptoms. A secondary aim was to explore the possible influence of between-study differences in psychotherapeutic framework, treatment format, intervention dose, cancer type, patient characteristics, study design, and risk of bias. Finally, to explore current developments in the field, we aimed to conduct a narrative evaluation of open trials (OTs) and noninferiority trials.

## METHODS

The current study was conducted in accordance with the Preferred Reporting Items for Systematic Reviews and Meta-Analyses (PRISMA) guidelines and was preregistered with PROSPERO (registration no.: CRD42017076514).^25^

### Search Strategy

We conducted keyword-based searches in PubMed, PsycINFO, Cochrane, CINAHL, and Embase databases. Keywords related to cancer (eg, neoplasm or oncology) were combined with keywords related to intervention (eg, psychotherap* or “cognitive-behav* therap*” or “psychol* treatment”) and terms related to fear (anxiet* or worr* or fear* or concern) and recurrence (relapse or recur* or progress*). The full search string is shown in the Data Supplement. Searches were conducted for the period from the earliest time available until June 2018, together with backward searches (snowballing) of reference lists of identified articles and earlier systematic reviews and forward searches (citation tracking).

### Selection Procedure and Data Extraction

English language reports published in peer-reviewed sources were included. We assessed study eligibility using the PICO approach (population, intervention, comparison, and outcome).^26^

Population: adult patients with or survivors of cancer (age 18 years or older). Studies of children and adolescents with cancer, patients without current cancer or a cancer history, or caregivers of patients with cancer were excluded.
Intervention: any psychological intervention that consisted primarily (> 50%) of psychological methods—for example, cognitive-behavioral, psycho-educative, imagery-based, and meditative approaches. Interventions that involved physical approaches—for example, yoga or exercise—could be included in the intervention but only if they were a secondary component (< 50%). Interventions were not required to directly target FCR.
Comparison: Eligible studies were required to use a control group—for example, waitlist, treatment as usual, or attention/active control. Case studies, studies that included only two active psychological interventions and no control group (eg, noninferiority trials), and open trials that employed uncontrolled pre–post designs were excluded from the meta-analysis. OTs, however, were included in the narrative systematic review.
Outcome: pre- and postintervention data, or pre–post change score data on one or more quantitative FCR-relevant construct. FCR could be both primary and secondary outcome. Only measures that pertained to concerns about the return or progression of cancer were included. Studies that used qualitative assessments, quantitative measures at one time point only, or only measures of general anxiety or worry were excluded. Studies needed to report results as either pre–post means and standard deviation/SE in all groups, change scores in all groups, effect sizes (ESs; eg, Cohen’s *d* or eta^2^), or provide other data that could be converted to an ES.

One author removed duplicates (A.B.S.) and five authors (N.M.T., J.G., A.B.S., B.T., and S.S.) took turns in pairs, each screening one third of the records and ensuring that all records were independently evaluated by two authors. Full texts of the remaining references were evaluated and reasons for exclusion registered (Data Supplement). Disagreements were discussed with a third author (N.M.T., B.T., or S.S.) until a negotiated conclusion was reached. Data were extracted by one author (N.M.T.) and checked by another author (C.M.). Studies were coded according to a priori–specified characteristics, including study, intervention, participant characteristics, and risk of bias.

### Computing ESs

Hedges’s *g*, a variation of Cohen’s *d*,^27^ correcting for possible bias as a result of small sample sizes,^28^ was used as the standardized between-group ES. Whenever possible, ESs were computed using means and their standard deviations for preintervention, postintervention, or change scores. If unavailable, ESs were estimated on the basis of other reported statistics—for example, *P* values, F values, or B values. Pooled ESs were weighted by the inverse SE, taking into account the precision of each study. The N used in the calculation was the N in the final analysis. A random effects model was chosen for all analyses, with positive values indicating ESs in the hypothesized direction. If studies reported results for more than one measure per outcome, the independence of results was ensured by averaging ESs across all outcomes so that only one result per study was used for each quantitative data synthesis.

### Heterogeneity

Heterogeneity was explored using Q and I^2^ statistics.^29^ Because of the generally low statistical power of heterogeneity tests, a more liberal *P* value of ≤ .10 was used to determine significant heterogeneity.^30^ The I^2^ statistic is an estimate of the variance in a pooled ES that is accounted for by heterogeneity in the sample of studies and is unaffected by the number of studies (K).^31^ I^2^ values of 0%, 25%, 50%, and 75% are taken to indicate no, low, moderate, and high heterogeneity, respectively.

### Publication Bias

Positive and negative findings are not equally likely to be published, and publication bias is a widespread problem when reviewing available evidence.^32^ We evaluated publication bias using funnel plots and Egger’s test.^33-35^ If results indicated possible publication bias, adjusted ESs were calculated using the Duval and Tweedie trim-and-fill method.^36^ In the case of statistically significant results, we calculated the failsafe number^33,37^ —that is, the number of unpublished studies with null findings that would reduce the results to statistical nonsignificance (*P* > .05)—and evaluated the robustness of results by comparing the failsafe number with the suggested criterion (5K + 10).^37^

### Risk of Bias Assessment

We adapted the Cochrane Collaboration tool^38^ to evaluate the risk of bias within the context of psychological intervention studies. We included the original domains of “random sequence allocation”, “allocation concealment”, “blinding of outcome assessment”, “accounting for attrition”, and “selective reporting”. We further differentiated “other sources of bias” with three subdomains: “treatment integrity” (ie, therapist training and fidelity), “conflict of interest” (ie, the trial was conducted by the therapists and/or the original developers of the therapy), and “bias in sampling and dropout” (eg, convenience sampling and uneven dropouts in intervention and control groups). Two authors (L.S. and G.O.) performed ratings independently. Disagreements were discussed with a third author (N.T.) until a negotiated final rating was reached for each study. Before the negotiation of a final rating, independent ratings were subjected to inter-rater reliability analyses (inter-rater agreement and κ statistics).^39^ Risk of bias scores were calculated for each study by evaluating the risk of bias for every item above as low, unclear (or not applicable), or high risk, rated as 0, 1, and 2, respectively. Associations between ESs and risk of bias scores were explored using meta-regression. Risk of bias scores were not used as weights when calculating aggregated ESs, as this is discouraged because of the risk of inducing bias.^40^

### Analytical Strategy

OTs and noninferiority trials were descriptively reviewed, and controlled trials (CTs) were subjected to meta-analysis to determine the pooled overall ES. Pooled ESs from baseline to post-treatment results and follow-up results were calculated separately. If multiple follow-up assessments were included, the longest follow-up assessment was chosen. Moderation analyses were performed with meta-regression on the basis of random-effects models and were estimated using the maximum likelihood method when data were available for 10 or more studies. Analyses were conducted using Comprehensive Meta-Analysis version 3 (http://www.meta-analysis.com).

We used the Grading of Recommendations Assessment, Development and Evaluation (GRADE) system^41^ to rate the quality of evidence of the meta-analytic results. Quality of evidence was graded as high, moderate, low, or very low. GRADE uses a baseline rating of high for RCTs and low for non-RCTs. This rating can be downgraded or upgraded on the basis of eight assessment criteria, including risk of bias, inconsistency of results, indirectness, imprecision, publication bias, effect magnitude, dose-response gradient, and the effect of all plausible confounding factors that would reduce the effect or suggest a spurious effect when no effect is found. Ratings were conducted and negotiated by two authors (M.S.O. and R.Z.).

## RESULTS

**FIG 1.**
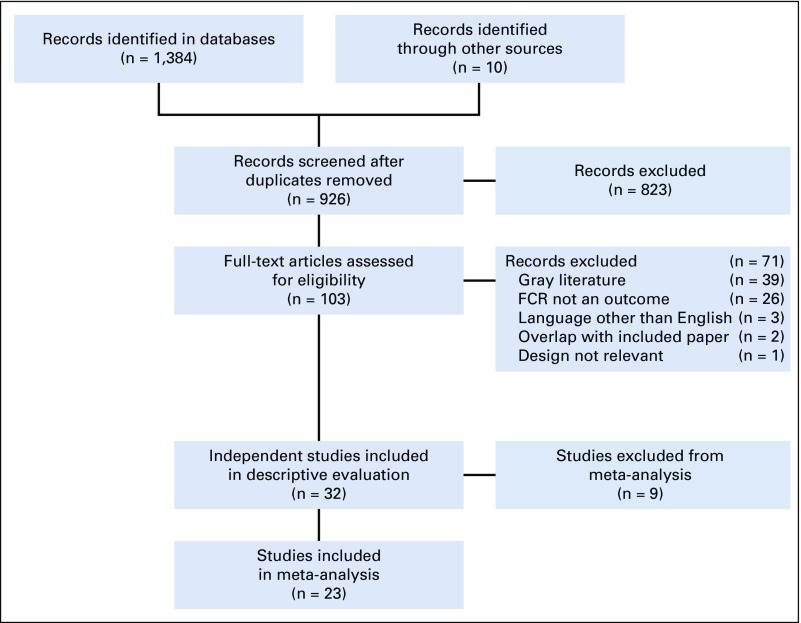
Study selection flowchart. FCR, fear of cancer recurrence.

The study selection process with reasons for exclusion is described in Figure 1 and the Data Supplement. The literature search yielded 1,394 references, of which 32 independent studies were subjected to descriptive evaluation. Of these, 23 CTs were subjected to meta-analytic evaluation.

### CT Characteristics

Study characteristics are listed in Table 1. The 23 CTs included a total of 2,965 patients with a mean sample size of 129.^42-60,62-64^ Of these, 21 studies reported post-treatment data, with 16 of these reporting relevant follow-up data. Two additional studies reported long-term (follow-up) data only. Post-treatment data were analyzed for 2,163 participants. Follow-up data were obtained 29 weeks on average after intervention and were analyzed for 2,044 participants. Most studies were RCTs (K = 21), with most control groups receiving no therapist attention (K = 19). Of the eight studies with FCR as the primary target of the intervention, FCR severity was an inclusion criterion in four studies only. All but one study were conducted in Western countries, participants were predominantly white, and, in most studies, the majority of participants were women (K = 21). Breast cancer was the most frequent cancer diagnosis (K = 15) and, in the majority of studies (K = 18), participants had no evidence of disease.

**TABLE 1.**
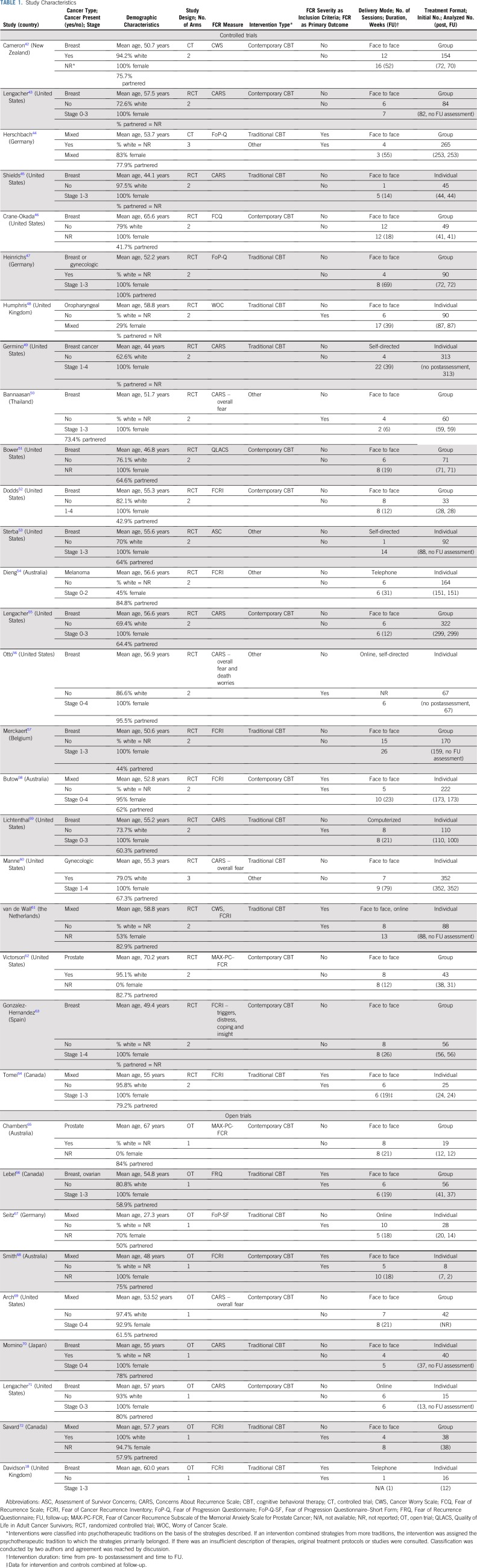
Study Characteristics

The 23 CTs evaluated a total of 25 interventions. Ten interventions used a traditional cognitive behavioral therapy (CBT) framework and nine interventions were contemporary CBTs. Studies were categorized as traditional CBT when interventions adhered to traditional cognitive behavioral principles that focus not only on Beckian therapy, but also on cognitive therapy principles that rely on information processing models in which the individual is assumed to hold biases, which gives rise to dysfunctional thoughts and beliefs.^73,74^ Contemporary CBTs were defined as interventions that were focused on the processes, rather than the content of cognition—for example, worry, rumination, attentional bias, and cognitive fusion—and aimed to change the way in which the individual related to his or her inner experiences.^75-77^ The remaining six interventions—other interventions—varied too much to be meaningfully grouped (eg, as psychodynamic therapy or supportive therapy). Approximately one half of interventions were group based (K = 13), with the remaining using an individual format (K = 12). In most studies, interventions were delivered face to face (K = 19). Number of sessions ranged from one to 15 (mean, 6.6). Reducing FCR was the primary aim in eight studies only.

### OT Characteristics

Nine OTs were eligible for descriptive evaluation (Table 1). All studies were described as feasibility or pilot studies and had sample sizes that ranged from eight to 56 (mean, 29.1). FCR severity was the inclusion criterion in three studies. Samples included prostate, breast, ovarian, and mixed types of cancer, with participants in three studies having current cancer. Five interventions could be categorized as traditional CBTs and the remaining four as contemporary CBTs. Five interventions had a primary aim of reducing FCR. Intervention was delivered in groups in four studies, all but three interventions were delivered face to face, and the number of sessions ranged from one to 10 (mean, 5.7). Eight studies reported positive statistically significant small-to-large within-subject ESs (range: Hedges’s* g* = 0.33-3.15).^18,65-67,69,71,72^ The remaining study found no statistically significant effect (*g* = 0.15; *P* = .44; no additional data shown).^70^

### Main Effects

**FIG 2.**
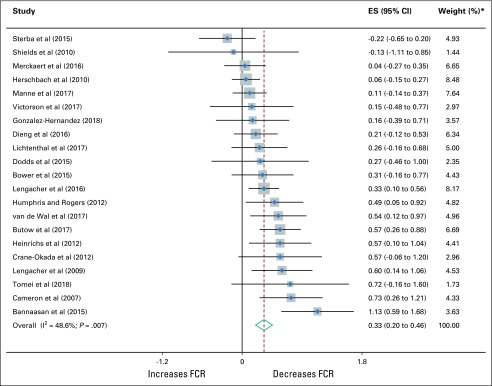
Forest plot of effect sizes (ESs; Hedges’s *g*) for effects at postintervention of psychological interventions on fear of cancer recurrence (FCR). (*) Weights are from random effects analysis.

Results of the meta-analyses are listed in Table 2 and illustrated with forest plots in Figure 2 and the Data Supplement. The overall combined postintervention ES was statistically significant and of small magnitude (*g* = 0.33; 95% CI, 0.20 to 0.46; *P* < .001). There were no indications of publication bias, and the failsafe number for effects at post-treatment (failsafe n = 255) exceeded the criterion (n = 115), which suggested a robust result. The overall combined effect at follow-up was statistically significant and only slightly smaller than at postintervention (*g* = 0.28; *P <* .001). Again, there were no indications of publication bias, and follow-up results seemed to be robust.

**TABLE 2.**
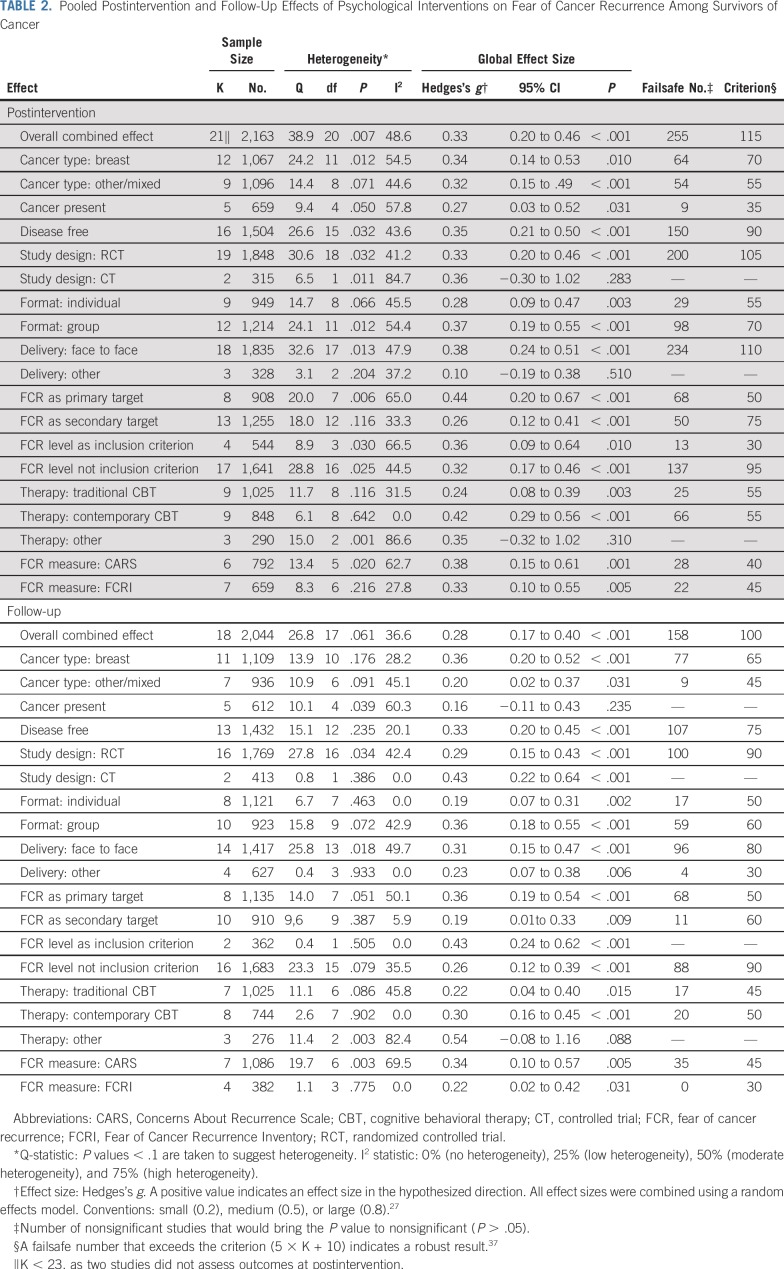
Pooled Postintervention and Follow-Up Effects of Psychological Interventions on Fear of Cancer Recurrence Among Survivors of Cancer

### Heterogeneity

Statistically significant Q tests and moderate I^2^ values for both postintervention (48.6%) and follow-up results (36.6%; Table 2) suggested some degree of variability in ESs beyond sampling error.

### Subgroup and Moderation Analyses

As shown in Table 2, when examining the results of the prespecified study subgroups—categorized according to cancer type, disease status, study design, format, delivery, FCR as primary or secondary target, FCR level as inclusion criterion or not, and psychotherapeutic framework—ESs were, with few exceptions, generally comparable across subgroups of studies. Almost all ESs were of small magnitude at both postintervention and follow-up. Results of the meta-regression analyses are listed in Table 3. At postintervention, effects of contemporary CBTs (*g* = 0.42) were larger than those of traditional CBTs (*g* = 0.24; β = .22; *P* = .018). At follow-up, larger effects were associated with shorter time to follow-up (in weeks; β = −.01; *P* = .027) and with group-based format compared with individual treatment format (β = .18; *P* = .041; Data Supplement). Changes in raw scores for the two most frequently used FCR measures—Concerns About Recurrence Scale and Fear of Cancer Recurrence Inventory—corresponded to mean differences of 1.3 (95% CI, 0.4 to 2.3; Concerns About Recurrence Scale overall fear) and 2.2 (95% CI, 1.4 to 3.1; Fear of Cancer Recurrence Inventory severity subscale; Data Supplement).

**TABLE 3.**
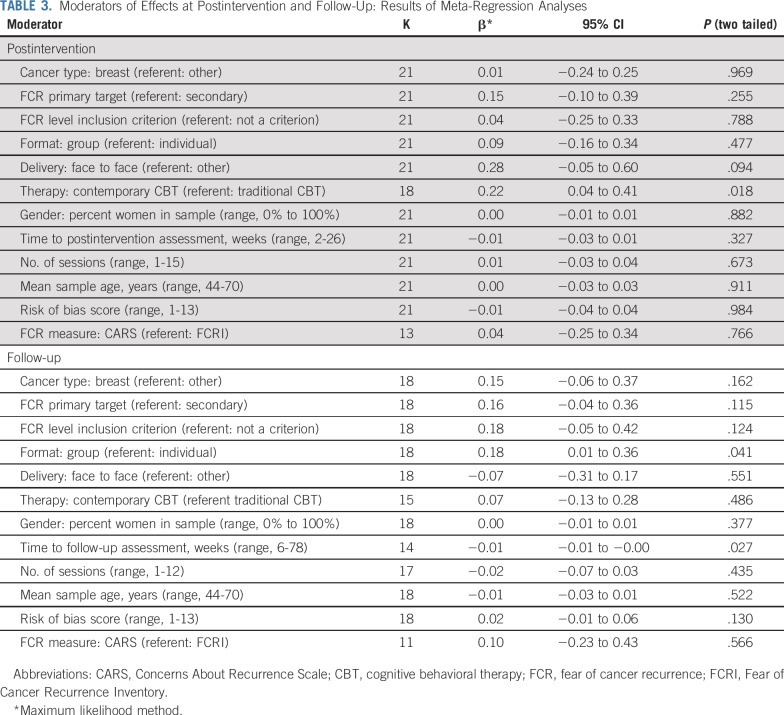
Moderators of Effects at Postintervention and Follow-Up: Results of Meta-Regression Analyses

### Risk of Bias

**FIG 3.**
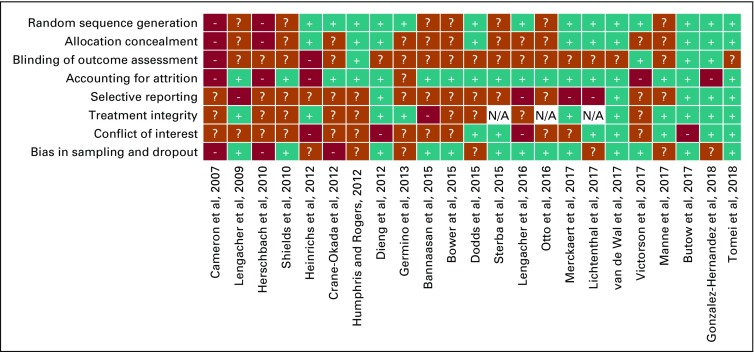
Risk of bias. Blue box with plus sign indicates a low risk of bias; red box with question mark indicates an unclear risk of bias; teal box with minus sign indicates a high risk of bias. N/A, not applicable.

Before negotiation, the two raters (L.S. and G.O.) agreed on 150 (81.5%) of 184 risk of bias ratings, and the inter-rater agreement (κ^39^) for the individual domains ranged from almost perfect (0.91; random sequence allocation) to fair (0.39; treatment integrity). Final negotiated results of risk of bias assessments for each study are shown in Figure 3 (for additional details, see the Data Supplement). No associations were found between total risk of bias scores and ESs at postintervention and follow-up (Table 3).

### Overall Quality of Meta-Analytic Evidence

The overall evidence for RCTs was qualified using GRADE.^41^ Overall, moderate quality of evidence demonstrates that psychological intervention may reduce FCR symptom compared with control conditions. The level of evidence for RCTs was downgraded to moderate as a result of concerns regarding inconsistency—that is, methodologic and clinical heterogeneity and inability to identify the reasons for heterogeneity—and indirectness—that is, that a considerable proportion of studies (K = 13) had FCR as secondary outcome, most studies included women only, and the majority of studies focused on FCR in cancer survivors, not fear of progression in patients with cancer present. Overall, no serious concerns were found for risk of bias, imprecision, and publication bias.

## DISCUSSION

The primary objective of the current study was to evaluate the efficacy of psychological interventions in alleviating FCR symptoms among patients with and survivors of cancer. Twenty-three controlled studies were identified, revealing a statistically significant effect on FCR outcomes of a small magnitude (*g* = 0.33) immediately after intervention, which was largely maintained at follow-up (*g* = 0.28), on average more than 7 months after the intervention. Results were robust with no indications of publication bias, which supported our hypothesis that psychological interventions would be efficacious relative to controls in reducing FCR symptoms. These findings are encouraging, given that managing FCR is a common unmet need among survivors of cancer^6^ and, when persistent and excessive, leaves the individual at risk of depression, impaired daily functioning, using unnecessary health assessments, and reduced quality of life.^1,2^ Furthermore, current findings point to lasting effects of FCR interventions beyond the immediate completion of the intervention. This finding is particularly relevant, as unmanaged FCR tends to stabilize over time.^2,7^ Here, it should be noted that follow-up times varied from 6 weeks to 78 weeks across studies and that meta-regression demonstrated that longer time to follow-up assessment was associated with a statistically significantly smaller effect. Number of sessions ranged from one to 15, with an average of 6.6 sessions, but no associations were found between the number of sessions and ES either at postintervention or at follow-up.

A secondary aim was to explore the possible influence of between-study differences. The larger effect found at postintervention for contemporary CBTs (*g* = 0.42) compared with traditional CBTs (*g* = 0.24) supports a hypothesis that FCR may be particularly responsive to contemporary therapies that aim to change the way in which individuals relate to their inner experiences by focusing on cognitive processing and metacognitions in FCR—for example, worry, rumination, or attentional bias.^78,79^ The difference no longer reached statistical significance at follow-up, mainly because of smaller ESs of contemporary CBTs at follow-up, which perhaps suggests that meta-cognitive skills learned in contemporary CBTs require booster sessions or materials to maintain long-term effects. Larger effects at follow-up were associated with shorter time to follow-up and with a group-based format compared with an individual treatment format. We have no clear explanation for the latter finding, which could be explored in future research.

All remaining moderation analyses failed to reach statistical significance. It has previously been found that newly diagnosed patients with cancer and younger survivors are more prone to experiencing high levels of FCR,^2^ but neither the presence of cancer, nor age was associated with overall intervention effect. Given the relatively small number of studies in the moderation analyses, which likely compromised our statistical power, two results should be noted when considering the numerical difference in ESs. First, the ES obtained at post-treatment with treatment delivered face to face was numerically larger (*g* = 0.38) than treatments that were delivered by other means (eg, telephone or Web based; *g* = 0.10). Only three studies used such other delivery means and results should be interpreted accordingly; the small number of studies demonstrated that delivery methods other than traditional face-to-face treatments are largely unexplored within the context of FCR. Internet-based interventions have previously been shown to be effective for anxiety disorders and fear-related conditions^80^ and have obtained equivalent effects to face-to-face treatments.^81^ It remains a question of whether this could be the case for FCR as well. Second, studies with FCR as their primary target obtained larger ESs at both postintervention and follow-up (*g* = 0.42; 0.36) than studies examining FCR as a secondary target (*g* = 0.26; 0.19). This finding should be further explored as the number of treatment studies increases, sufficiently powering analyses to test whether treatments with FCR as their primary target are superior in reducing FCR symptoms compared with generalized interventions. In addition, only four studies included participants on the basis of their FCR symptom levels and it is unclear to what degree this may have influenced results.

Robust but relatively small effects point to a number of potential implications, both clinically and for future research. Establishing the efficacy of psychological interventions for FCR should also concern which treatment components may be most efficacious or which processes drive the effect. Fardell et al^78^ have suggested a number of key maintaining processes of FCR, resulting in a theoretical model with dysfunctional cognitive processes at its core. The authors suggest that particular treatment components from contemporary CBTs, including metacognitive therapy^82^ and acceptance and commitment therapy,^83^ are well suited for targeting such processes. Future treatment trials should not only establish the efficacy of their treatment, but also investigate which components are most change potent. One approach could be the Multiphase Optimization Strategy,^84^ a systematic method for exploring the main and interactive effects of treatment components and investigating select treatment components in a factorial design where all possible combinations of components are evaluated. Furthermore, the dose needed for effective treatment of FCR is likely not identical for all individuals and intervention researchers are increasingly interested in ways to individually tailor psychotherapy (eg, Fisher and Boswell).^85^ Existing therapies already suggest conducting a thorough individual case formulation^58^; however, to date, treatment programs for FCR have not outlined or investigated markers—for example, time since diagnosis, severity of FCR, or level or type of dysfunctional cognitive processes—suggestive of including or abandoning certain treatment components or increasing or decreasing the dose. Theoretical formulations of FCR^78^ could guide researchers in identifying relevant markers to investigate.

Our results should be viewed in light of limitations that pertain to the methodology of the included studies and between-study heterogeneity, noting that overall strength of the evidence was downgraded to moderate. Many studies suffered from the risk of selective reporting. Although evaluating the effect within the different categories pertaining to each of the identified moderators, between-study heterogeneity for most categories remained moderate to large. This could indicate potentially unidentified variables that are responsible for systematic variation. Finally, it should be noted that all but four authors have contributed to the studies included in the present review, which might raise concerns regarding bias. However, this may be less of an issue as the review was preregistered; all authors agreed to the final protocol; the first, second, and corresponding authors (N.M.T., M.S.O., and R.Z.) have not yet published any intervention studies on FCR; and screening and data extraction was performed by authors who had not been principle investigators of any of the reviewed studies.

In conclusion, to our knowledge, this is currently the most comprehensive systematic review and meta-analysis of the effect of controlled psychological intervention studies specifically on FCR outcomes. Twenty-three CTs were located, revealing a statistically significant effect on FCR outcomes of a small magnitude that was largely maintained at follow-up. Psychological interventions therefore seem to be efficacious in reducing FCR symptoms. Future trials should focus on targeted interventions for FCR, include participants on the basis of high levels of FCR, and investigate how to further optimize interventions—for instance, by exploring the effect of different treatment components and tailoring the intervention to the individual’s FCR symptoms.
